# Editorial: Sexual and Parasexual Reproduction of Human Fungal Pathogens

**DOI:** 10.3389/fcimb.2022.934267

**Published:** 2022-06-28

**Authors:** Ulrich Kück, Richard J. Bennett, Linqi Wang, Paul S. Dyer

**Affiliations:** ^1^ Allgemeine & Molekulare Botanik, Ruhr-University, Bochum, Germany; ^2^ Department of Molecular Microbiology and Immunology, Brown University, Providence, RI, United States; ^3^ State Key Laboratory of Mycology, Institute of Microbiology, Chinese Academy of Sciences, Beijing, China; ^4^ School of Life Sciences, University of Nottingham, Nottingham, United Kingdom

**Keywords:** human fungal pathogens, propagation, sexual cycles, genetics, fungal reproduction

In recent years, human fungal pathogens have received increasing attention, with several of these species responsible for prevalent diseases that are associated with high mortality rates. The major human pathogens belong to the genera *Candida*, *Aspergillus*, and *Cryptococcus*, although a wide variety of highly diverse fungal species are clinically relevant. This increased interest has arisen due to a number of factors such as the presence of ever higher numbers of clinically vulnerable patients who are susceptible to fungal infections, better identification and diagnosis of fungal diseases, and the emergence and spread of isolates showing resistance to commonly used antifungal drugs. It is therefore of both fundamental interest and medical importance to understand how human fungal pathogens can generate variation and evolve over time.

Historically, most human fungal pathogens were thought to propagate primarily through clonal asexual reproduction. During this process, cells can still accumulate mutations and can undergo a variety of genomic rearrangements and ploidy alterations. Such changes can contribute to adaptive changes associated with enhanced pathogenicity, resistance to antifungal drugs, and persistence in chronic infections. Two reviews summarize our current view on parasexual propagation in various *Candida* species.

Mishra et al., (2021) provide a comprehensive review of current understanding of parasexual reproduction, the underlying molecular mechanisms governing its regulation, and its relevance to *Candida* biology. The article reminds us that the discovery of parasexuality in *Candida albicans* roughly two decades ago has served as a model for other *Candida* species for which sexual cycles have not been described. In particular, parallels between parasexual cycles in *C. albicans* and meiotic divisions in other fungi are emphasized, and their relevance to natural populations are illustrated.

Xu (2021) addresses the question of how genetic diversity is generated in natural populations of *Candida tropicalis*. The article reviews current understanding of the potential modes of reproduction in this species and describes the expectations of modes of sexual, parasexual, and asexual reproduction, comparing them with the patterns of genetic variation observed in natural populations.

Full sexual cycles can also impact the evolution of important fungal attributes such as pathogenicity and antifungal drug resistance, as well as provide a valuable tool for genetic analysis. Although long overlooked, discoveries of complete sexual cycles in species such as *Aspergillus fumigatus* have widened our understanding of how sex occurs and how it may contribute to pathogenesis. Such discoveries are greatly assisted by insights from pioneering genome sequencing projects in the early 2000s and followed previous studies that provided indirect evidence for sexual reproduction in natural populations.

The sexual cycle of *A. fumigatus* had previously only been demonstrated under laboratory conditions and there has been much speculation as to if and where mating might occur in nature. Zhang et al., (2021) demonstrate that flower bulb waste material from stockpiles undergoing composting can provide the environmental conditions necessary for sexual reproduction in *A. fumigatus*. Specialist membranes and fluorescent stains were used to demonstrate the presence of sexual ascospores within the waste material and it was further shown that ascospores can germinate after exposure to 70°C for periods of up to several days in contrast to asexual conidiospores, which lost viability. Finally, it was confirmed that flower bulb waste material could serve as a natural substrate for sex by the demonstration that such material could be utilized successfully to perform sexual crosses in the laboratory.

Ashton et al., (2022) also demonstrate that the sexual cycle can be used for genetic analysis. They used the ‘bulk segregant analysis (BSA)’ technique together with next-generation sequencing and a newly developed bioinformatic pipeline to investigate the underlying basis of monogenic traits in *A. fumigatus*. Using a clinical isolate exhibiting monogenic resistance to the azole antifungal itraconazole in a cross with a sensitive partner strain, they identified 123 genes in F1 progeny that are potentially responsible for resistance to itraconazole. They then used a combination of backcrossing and an increased number of progeny in the BSA pools to narrow this down to just 20 candidate genes and were then able to that show a change of methionine to lysine codon in the *cyp51A* gene was responsible for antifungal resistance. This study shows great promise for future applications of BSA in *A. fumigatus* and beyond.

Sexual reproduction is also evident in the opportunistic yeast-like pathogen *Cryptococcus neoformans*, where it can promote the generation of hyper-virulent and drug-resistant strains and produces infectious meiospores. The article by Wu et al., (2022) addresses the question of how the ubiquitin-proteasome system regulates sexual reproduction in *C. neoformans*. The article revealed that the F-box protein Cdc4 is critical for fungal virulence and sexual reproduction in *C. neoformans*. In particular, Cdc4 regulates cell membrane integrity and repair of DNA damage in this pathogen. Moreover, when used in bilateral mating experiments, *cdc4* deletion mutants showed a block in their ability to undergo meiosis after nuclear fusion within the basidia, ultimately failing to form meiospores.

In conclusion, the articles and reviews in this Research Topic expand our understanding of how sexual and parasexual reproduction of human fungal pathogens can drive the adaptive genetic changes that contribute to key traits, including those impacting antifungal drug resistance and pathogenesis in the mammalian host. They also highlight how sexual reproduction provides powerful tools with which to genetically dissect key aspects of fungal biology.

## Author Contributions

All authors listed have made a substantial, direct, and intellectual contribution to the work and approved it for publication.

## Conflict of Interest

The authors declare that the research was conducted in the absence of any commercial or financial relationships that could be construed as a potential conflict of interest.

## Publisher’s Note

All claims expressed in this article are solely those of the authors and do not necessarily represent those of their affiliated organizations, or those of the publisher, the editors and the reviewers. Any product that may be evaluated in this article, or claim that may be made by its manufacturer, is not guaranteed or endorsed by the publisher.
